# Prebiotics, Probiotics, and Gut Microbiota with Chronic Disease (2nd Edition): Advances, Challenges, and Future Perspectives

**DOI:** 10.3390/nu18121876

**Published:** 2026-06-10

**Authors:** Qingsen Shang

**Affiliations:** 1 Key Laboratory of Marine Drugs of Ministry of Education, Shandong Key Laboratory of Glycoscience and Glycotherapeutics, School of Medicine and Pharmacy, Ocean University of China, Qingdao 266003, China; shangqingsen@ouc.edu.cn or shangqingsen@163.com; 2 Laboratory for Marine Drugs and Bioproducts, Qingdao Marine Science and Technology Center, Qingdao 266237, China; 3 Marine Biomedical Research Institute of Qingdao, Qingdao 266071, China

## 1. Introduction

Chronic diseases, including cardiovascular disease, type 2 diabetes mellitus (T2DM), obesity, inflammatory bowel diseases (IBD), chronic kidney disease (CKD), cancer, and autoimmune disorders, represent the leading causes of mortality and morbidity worldwide [1]. Over the past two decades, the gut microbiota has emerged as a critical modulator of host physiology, profoundly influencing metabolic homeostasis, immune function, and inflammatory processes [2,3,4]. The recognition that gut dysbiosis contributes to the pathogenesis and progression of chronic diseases has catalyzed intensive research into microbiome-targeted interventions, including prebiotics, probiotics, synbiotics, postbiotics, and live biotherapeutic products [5,6,7,8].

## 2. Overview of the Special Issue Contributions

The 13 articles published in this Special Issue can be broadly categorized into three thematic areas: (i) novel synbiotic formulation, probiotic strains, and mechanistic investigations; (ii) comprehensive reviews on disease-specific microbiome–host interactions; and (iii) novel concepts and perspectives on microbiome-targeted nutrition.

### 2.1. Novel Synbiotic Formulation, Probiotic Strains, and Mechanistic Investigations

In this Special Issue, Luo et al. provide a timely and mechanistically grounded study demonstrating that a synbiotic formulation combining the human milk oligosaccharide lacto-*N*-tetraose (LNT) with *Bifidobacterium animalis* subsp. *lactis* MN-Gup confers superior metabolic benefits over monotherapies in a high-fat diet-induced obese mouse model. Notably, the high-dose synbiotic intervention not only attenuated weight gain, glucose intolerance, and hepatic steatosis but also restored intestinal barrier integrity and remodeled the gut microbiota toward a healthier profile. This work highlights the potential of rationally designed synbiotics to target the gut–liver axis and suggests that LNT may serve as a selective prebiotic to enhance the in vivo efficacy of probiotics.

The study by Wei et al. presents a compelling investigation of *Lactiplantibacillus plantarum* WLPL04, a strain isolated from human breast milk, in a murine model of hyperuricemia (HUA). This work exemplifies the multi-targeted therapeutic potential of next-generation probiotics. The authors demonstrated that *L. plantarum* WLPL04 attenuates HUA through three coordinated mechanisms: (i) modulation of host purine metabolism via upregulation of salvage pathway genes (*hpt* and *xpt*); (ii) regulation of renal urate transporters (upregulation of ABCG2 and downregulation of URAT1 and GLUT9); and (iii) remodeling of gut microbiota composition with enrichment of beneficial *Bacteroides* species. This study is particularly significant as it moves beyond simple correlation to elucidate strain-specific molecular mechanisms linking gut microbiota modulation with host metabolic and inflammatory pathways.

Two complementary studies in this Special Issue advance our understanding of pro-biotic-mediated protection against IBD. The investigation by Dai et al. introduces *Bacteroides salyersiae* CSP6 as a candidate next-generation probiotic species with potent anti-colitis properties. This work is notable for several reasons: first, it provides the first in vivo evidence for the protective role of *B. salyersiae*, a commensal species previously underexplored in disease contexts; second, it demonstrates that the protective effects are mediated not only by direct bacterial interactions but also by metabolite-driven mechanisms, specifically through the enrichment of equol, 8-deoxylactucin, and tiglic acid—metabolites with established anti-inflammatory properties; and third, it highlights the importance of ecological competition, showing that *B. salyersiae* CSP6 effectively suppresses pathogenic *Escherichia-Shigella* spp. while promoting beneficial *Dubosiella* spp. and *Bifidobacterium pseudolongum*. This study reinforces the paradigm that next-generation probiotics should be selected based on their functional ecological roles rather than merely on their taxonomic identity.

In parallel, Deleu et al. report an elegant study employing engineered *Saccharomyces cerevisiae* var. *boulardii* strains with enhanced acetate production in a dextran sodium sulfate (DSS)-induced colitis model. Their findings establish a potential dose–response relationship between acetate production capacity and anti-inflammatory efficacy, with high-acetate-producing strains significantly reducing disease activity and histological inflammation compared to non-acetate-producing or transient-acetate-producing controls. Transcriptomic analysis further corroborated these findings at the molecular level. This work provides pre-clinical rationale for the development of metabolically engineered probiotics and underscores the critical role of short-chain fatty acids (SCFAs), particularly acetate, in maintaining intestinal homeostasis. Together, these two studies illustrate the evolving landscape of probiotic research—from traditional strains to genetically defined, functionally optimized live biotherapeutics.

Maćków et al. conducted a two-center pilot study examining dietary supplement use among Polish children with IBD. While this study does not directly investigate microbiome modulation, it provides crucial clinical context by revealing that 81.4% of parents of children with IBD use dietary supplements, primarily based on physician recommendation. The study identified significant associations between supplementation patterns, disease stage, and nutritional status, highlighting the importance of vitamin D, B6, E, and iron supplementation in this vulnerable population. This work serves as an important reminder that microbiome-targeted interventions must be integrated within broader nutritional management strategies, particularly in pediatric populations.

### 2.2. Comprehensive Reviews on Disease-Specific Microbiome–Host Interactions

Smółka et al. present a meticulously structured review examining the emerging gut microbiome–venous thromboembolism (VTE) axis. The authors critically evaluate the mechanistic links between gut dysbiosis, endotoxemia, microbial metabolites, and thromboinflammation. Their analysis reveals that while the biological plausibility of this axis is well-supported, direct human VTE-specific evidence remains limited and heterogeneous. Microbiome-derived metabolites such as trimethylamine *N*-oxide (TMAO), phenylacetylglutamine (PAGln), bile acids, and SCFAs are implicated in thrombosis-related biology, yet most evidence derives from experimental or non-VTE settings. This review is exemplary in its cautious interpretation of associative data and its clear delineation of the need for longitudinal and mechanistically informed studies.

Di Renzo et al. provide a comprehensive critical evaluation of clinical trial evidence for probiotics, prebiotics, and synbiotics in CKD management. The authors systematically demonstrate that while these interventions can promote beneficial shifts in gut microbiota composition, enhance saccharolytic fermentation, increase SCFA production, and reduce circulating uremic toxins (indoxyl sulfate and *p*-cresyl sulfate), the evidence for improvements in renal function and hard clinical outcomes remains inconclusive. Key limitations identified include heterogeneity in study design, probiotic strains, prebiotic substrates, dosing regimens, and patient populations, compounded by small sample sizes and short intervention durations. Notably, the review finds that synbiotics do not consistently demonstrate superior clinical efficacy over individual components, challenging theoretical assumptions about synergistic effects. This balanced assessment is essential for guiding future trial design and managing clinical expectations.

In a second contribution, Smółka et al. synthesize evidence linking gut microbiota to aortic diseases, including abdominal aortic aneurysm (AAA), thoracic aortic aneurysm (TAA), aortic dissection (AD), and Takayasu arteritis (TAK). The strongest evidence is identified for AAA, where multiple cohorts report gut dysbiosis and reduced microbial diversity, and translational studies have detected bacterial DNA and microbial products in blood, aneurysm walls, and intraluminal thrombus. Microbiota-derived mediators, including TMAO, lipopolysaccharides (LPS), SCFAs, and bile acid derivatives, interact with pathways governing cytokine signaling, oxidative stress, innate immune activation, and extracellular matrix degradation. However, the authors appropriately caution that low-biomass findings do not establish microbial viability or causality, and Mendelian randomization analyses yield heterogeneous results. This review highlights the need for longitudinal multi-compartment studies and targeted interventions with aortic-specific endpoints.

Park provides a focused review on the role of *Lactobacilli* strains in cancer management, synthesizing studies from 2021 to 2025. The review documents strain-specific and cancer-type-dependent tumor-suppressive activities mediated through apoptosis induction, migration inhibition, and regulation of oncogenic signaling pathways. Additionally, *Lactobacilli* demonstrate immune-enhancing properties and capacity to reshape gut microbiota balance. The author appropriately identifies current limitations in research methodologies, including lack of standardization in strains, doses, and treatment regimens, and proposes avenues for future investigation. This review is timely given the growing interest in microbiome-modulatory strategies as adjuncts to conventional cancer therapy.

Kleniewska and Pawliczak present an integrative review linking gut and airway dysbiosis, inflammation, oxidative stress, and asthma pathogenesis. The authors argue that modern lifestyle factors contribute to dysbiosis and pro-oxidant–antioxidant imbalance, thereby exacerbating asthma susceptibility and severity. They propose that probiotics, prebiotics, and antioxidants represent promising complementary strategies for asthma management through immune homeostasis maintenance, anti-inflammatory effects, and oxidative stress mitigation. This review effectively bridges the gut–lung axis concept with practical nutritional interventions.

Iatcu et al. deliver an Editor’s Choice review examining the impact of diverse prebiotics, including inulin, fructooligosaccharides, galactooligosaccharides, resistant starch, pectic oligosaccharides, polyphenols, β-glucan, and *Dendrobium officinale*, on T2DM outcomes. The authors comprehensively document how these prebiotics improve glycemic control while promoting beneficial bacteria, particularly butyrate producers such as *Roseburia* spp. and *Faecalibacterium* spp., which are typically depleted in T2DM patients. The review advances the concept of personalized dietary interventions based on individual microbiota profiles, representing a significant step toward precision nutrition in diabetes management.

Bui provides a conceptual review positioning the human microbiome as a distinct organ in symbiotic relationship with the host. The author discusses how industrial lifestyles, which are characterized by processed food consumption, alcohol use, and antibiotic exposure, have unfavorably altered the gut microbiome, contributing to metabolic diseases. This review serves as a foundational framework for understanding microbiome-targeted therapeutic strategies in metabolic health.

### 2.3. Novel Concepts and Perspectives on Microbiome-Targeted Nutrition

Yang et al. contribute a thought-provoking perspective proposing that exogenous proteases and lipases may function as prebiotics. Reviewing 13 studies, the authors demonstrate that dietary supplementation with these digestive enzymes consistently im-proves animal gut microbiota composition and increases beneficial bacteria and SCFA levels. The proposed mechanism involves enhanced nutrient digestion increasing substrate availability for beneficial microbes. This represents a paradigm expansion in prebiotic definition and suggests that digestive enzymes, already widely used in food production and animal nutrition, may have underappreciated roles in human gut health. This perspective invites further investigation into the prebiotic-like effects of exogenous enzymes and their potential applications in chronic disease management.

## 3. Synthesis and Critical Assessment

Collectively, the contributions to this Special Issue reveal several important themes and advances in the field:

First, the field is transitioning from descriptive association studies to mechanistic investigations. The original research articles exemplify this shift by elucidating how probiotics exert their effects through specific molecular pathways such as purine metabolism, renal transporter regulation, inflammasome modulation, and SCFA-mediated anti-inflammatory signaling. This mechanistic depth is essential for advancing beyond proof-of-concept to rational therapeutic design.

Second, novel synbiotic formulation, next-generation probiotics, and engineered live biotherapeutics represent the frontier of microbiome medicine. The studies on LNT with *B. animalis* subsp. *lactis* MN-Gup, *B. salyersiae* CSP6, and acetate-producing *S. cerevisiae* var. *boulardii* demonstrate that strain selection and metabolic engineering can optimize therapeutic efficacy. However, significant challenges remain in translating these preclinical findings to human applications, including safety assessment, regulatory pathways, manufacturing scalability, and long-term colonization dynamics.

Third, the reviews collectively highlight a critical gap between promising preclinical and early clinical data and robust evidence for hard clinical outcomes. In conditions ranging from CKD, VTE, and aortic diseases to cancer, microbiome-targeted interventions frequently improve surrogate biomarkers such as microbiota composition, SCFA levels, inflammatory markers, and uremic toxin concentrations. Nevertheless, these improvements have not consistently translated into demonstrable benefits in disease progression, complication rates, or mortality. This discrepancy underscores the need for larger, longer-duration, multicenter randomized controlled trials with standardized formulations and clinically meaningful endpoints.

Fourth, the concept of precision microbiome medicine is gaining traction. The recognition that probiotic effects are strain-specific and disease-dependent, and that prebiotic responses vary with baseline microbiota composition, necessitates personalized approaches. The review by Iatcu et al. on prebiotics in T2DM explicitly advocates for personalized dietary interventions, reflecting a broader trend toward individualized microbiome modulation.

Fifth, the gut–organ axis framework continues to expand. The contributions on VTE, aortic diseases, obesity, non-alcoholic fatty liver disease, CKD, and asthma illustrate how gut dysbiosis influences distant organ systems through diverse mechanisms. Among these, microbial translocation, circulating metabolites, immune modulation, and systemic inflammation play central roles. This systems-level perspective is essential for understanding the pleiotropic effects of microbiome-targeted interventions.

## 4. Future Directions and Emerging Challenges

Based on the contributions to this Special Issue and the broader evolving landscape, I identify several priority areas for future research:

### 4.1. Standardization and Clinical Validation

A paramount challenge is the lack of standardization in microbiome-targeted intervention trials. Future studies must adopt harmonized protocols for strain selection, dosing, duration, outcome assessment, and microbiome analysis. The development of qualified biomarkers that reliably predict clinical response to microbiome modulation is urgently needed. Additionally, regulatory frameworks for live biotherapeutic products need refinement to accommodate the unique characteristics of microbiome-based therapies.

### 4.2. Mechanistic Elucidation and Multi-Omics Integration

While significant progress has been made in identifying individual mechanisms, the complex, multi-scale interactions within the microbiome–host ecosystem require integrated multi-omics approaches. Future studies should combine metagenomics, metatranscriptomics, metabolomics, proteomics, and host transcriptomics to construct comprehensive interaction networks. Advanced computational methods, including machine learning and artificial intelligence, will be essential for integrating these complex datasets and predicting therapeutic outcomes.

### 4.3. Next-Generation Prebiotics and Postbiotics

The Special Issue contributions primarily focus on traditional prebiotics and live probiotics. However, the field is rapidly expanding toward next-generation prebiotics (targeted glycans, human milk oligosaccharides, microbial growth factors) and postbiotics (inactivated microbial cells, cell fractions, secreted metabolites). These approaches offer potential advantages in safety, stability, and regulatory compliance. The perspective by Yang et al. on digestive enzymes as new sources of prebiotics exemplifies the expanding conceptual boundaries of microbiome modulation.

### 4.4. Fecal Microbiota Transplantation and Defined Consortia

While not extensively covered in this Special Issue, fecal microbiota transplantation (FMT) and defined microbial consortia represent important therapeutic modalities for chronic diseases. Future research should focus on developing standardized, characterized consortia with defined therapeutic mechanisms, moving beyond the crude complexity of whole fecal transplants. The principles of rational design of functionally defined therapeutics, as demonstrated by engineered *S. cerevisiae* studies, should guide this evolution.

### 4.5. Longitudinal and Life-Course Perspectives

Chronic diseases develop over years to decades, yet most intervention studies are limited to weeks or months. Longitudinal cohort studies tracking microbiome dynamics from early life through disease onset and progression are essential for identifying critical windows for intervention. The pediatric IBD study by Maćków et al. highlights the im-portance of early-life nutritional and microbiome considerations.

### 4.6. Integration with Conventional Therapies

Microbiome-targeted interventions should not be viewed as replacements for conventional therapies but as complementary strategies. Future research must rigorously evaluate interactions between microbiome modulators and standard-of-care treatments (e.g., immunosuppressants in IBD, chemotherapy in cancer, statins in cardiovascular disease) to optimize combination regimens and avoid adverse interactions.

### 4.7. Global and Environmental Context

The reviewed studies predominantly reflect populations in Europe, North America, and East Asia. Given the substantial geographic and cultural variation in dietary patterns, microbiota composition, and chronic disease prevalence, research in underrepresented populations is critical. Additionally, environmental factors, including pollution, climate change, and antibiotic stewardship, profoundly influence the microbiome and must be incorporated into holistic disease prevention strategies.

## 5. Conclusions

This second edition of the Special Issue “Prebiotics, Probiotics, and Gut Microbiota with Chronic Disease” advances our understanding of the multifaceted relationships be-tween the gut microbiome and chronic disease pathogenesis. It also highlights both the tremendous potential and significant challenges of microbiome-targeted therapeutics. The published contributions span from molecular mechanistic studies to comprehensive clinical reviews, from novel probiotic discovery to conceptual expansions of prebiotic definitions, collectively painting a picture of a field in dynamic evolution.

As Guest Editor, I am deeply grateful to all contributing authors for their rigorous scholarship and innovative research, to the reviewers for their critical assessments that ensured publication quality, and to the editorial team at *Nutrients* for their professional support throughout this process. The collective efforts reflected in this Special Issue bring us closer to realizing the promise of microbiome medicine: the development of safe, effective, and personalized interventions that harness the power of our microbial symbionts to prevent and treat chronic diseases.

The road ahead requires continued mechanistic investigation, rigorous clinical validation, methodological standardization, and interdisciplinary collaboration. Yet, as the contributions to this Special Issue demonstrate, we are making tangible progress toward translating microbiome science into meaningful clinical benefits for patients worldwide.
